# Searching for Synergistic Bronchodilators and Novel Therapeutic Regimens for Chronic Lung Diseases from a Traditional Chinese Medicine, Qingfei Xiaoyan Wan

**DOI:** 10.1371/journal.pone.0113104

**Published:** 2014-11-14

**Authors:** Yuanyuan Hou, Binfeng Cheng, Mengge Zhou, Runping Fang, Min Jiang, Wenbin Hou, Gang Bai

**Affiliations:** 1 State Key Laboratory of Medicinal Chemical Biology and College of Pharmacy, Tianjin Key Laboratory of Molecular Drug Research, Nankai University, Tianjin, China; 2 Tianjin Engineering Laboratory of Quality Control Techniques for TCM, Tianjin Institute of Pharmaceutical Research, Tianjin, China; Macau University of Science and Technology, Macao

## Abstract

Classical Chinese pharmacopeias describe numerous excellent herbal formulations, and each prescription is an outstanding pool of effective compounds for drug discovery. Clarifying the bioactivity of the combined mechanisms of the ingredients in complex traditional Chinese medicine formulas is challenging. A classical formula known as Qingfei Xiaoyan Wan, used clinically as a treatment for prevalent chronic lung disease, was investigated in this work. A mutually enhanced bioactivity-guided ultra-performance liquid chromatography/quadrupole time-of-flight mass spectrometry (UPLC/Q-TOF-MS) characterization system was proposed, coupled with a dual-luciferase reporter assay for β_2_AR-agonist cofactor screening. Arctiin, arctigenin, descurainoside and descurainolide B, four lignin compounds that showed synergistic bronchodilation effects with ephedrine, were revealed. The synergistic mechanism of arctigenin with the β_2_ARagonist involved with the reduction of free Ca^2+^ was clarified by a dual-luciferase reporter assay for intracellular calcium and the Ca^2+^ indicator fluo-4/AM to monitor changes in the fluorescence. The relaxant and contractile responses of airway smooth muscle are regulated by crosstalk between the intracellular cAMP and calcium signaling pathways. Our data indicated the non-selective βAR agonist ephedrine as the principal bronchodilator of the formula, whereas the lignin ingredients served as adjuvant ingredients. A greater understanding of the mechanisms governing the control of these pathways, based on conventional wisdom, could lead to the identification of novel therapeutic targets or new agents for the treatment of asthma and COPD.

## Introduction

In recent decades, biotechnology has provided novel approaches to drug development and generated new classes of biological therapeutics. Researchers have focused on drug discovery, using an important group of complementary and alternative therapeutics, herbal medicines and botanical sources [1], [2]. Traditional Chinese medicines (TCMs) have attracted increased global attention because they are outstanding pools of effective compounds for drug discovery with long clinical use and reliable therapeutic efficacy [3]. Classical Chinese pharmacopeias describe many excellent herbal formulations used to treat various diseases, particularly chronic conditions [4]. TCM formulas are prescribed so that each herb is used to its greatest potential, thus improving the treatment results and reducing any adverse effects caused by combined herbal drugs [5]. The efficacy of TCM is attributed to the complex mixture of chemical compounds present in the various herbs. The principal ingredient is the substance that provides the main therapeutic effect and is included in the monarch drugs. The second principal ingredient enhances or assists the actions of the first and is generally a minister or assistant drug [6]. Combinatorial medicines have been gaining acceptance in the West, and examples of these combinations include the drug cocktails used to treat acquired immunodeficiency syndrome and cancer, as well as complex antibiotic medications [7], [8]. These therapeutics contain a limited number of pure compounds that have been well characterized. TCMs are notably more challenging because of the variability of the individual herbs and the chemical complexities of the formulations. Isolation methodologies and the characterization of bioactive compounds from plant resources have recently undergone rapid development [9], [10]. Combined with a target-based reporter gene assay, bioactivity-guided ultra-performance liquid chromatography/quadrupole time-of-flight mass spectrometry (UPLC/Q-TOF-MS) has been applied to screen receptor agonists or inhibitors from botanical drugs [11], [12]. Compared to conventional methods, this powerful tool facilitates the screening and identification of potential lead compounds in complex herbal extracts. The synergistic interaction between TCM ingredients has not been revealed by these strategies.

Asthma and chronic obstructive pulmonary disease (COPD) are the two most common chronic lung diseases worldwide, and they could become the third leading cause of death by 2030 [13], [14]. The pharmacological treatments, as summarized in the Global Initiative for Chronic Obstructive Lung Disease (GOLD) guidelines for managing stable COPD, include bronchodilators, β_2_-adrenergic receptor (β_2_AR) agonists and inhaled glucocorticosteroids [15]. Modulating multiple biological targets together, rather than regulating a single-target, could be beneficial for the treatment of diseases with complex etiologies. Concurrently, complementary and alternative therapies are increasingly important in treating complex diseases because they could act on multiple targets in the disease network [16], [17]. TCMs differ from therapeutics based on single-chemical entities. A number of herbal medicines and ancient TCM prescriptions are novel therapeutic regimens against COPD or asthma [18]–[21]; naturalβ_2_AR agonists or antagonists participate in the regulation process, and TCM prescription compatibility enhances the regulation pathway more effectively [22].

Qingfei Xiaoyan Wan (QFXY) evolved from a classical TCM prescription that has been used to treat pulmonary diseases since 200 B.C., known as Maxing Shigan decoction. QFXY consists of the following eight herb preparations: Ephedra Herba as a monarch drug, Saigae Tataricae Cornu, Pheretima, Arctii Fructus, Lepidii Semen, Bovis Calculus Artifactus, Armeniacae Semen Amarum, and Gypsum Fibrosum as minister or assistant drugs. In clinical practice, QFXY has a good clinical therapeutic effect against COPD, asthma, and lung inflammation because it relieves various respiratory symptoms. In a previous work, a UPLC/Q-TOF-MS system using two-cell-based dual-luciferase reporter assays was established to screen for NF-κB inhibitors and β_2_AR agonists, and four types of compounds (arctigenin derivatives, cholic acid derivatives, chlorogenic acid, and sinapinic acid) with anti-inflammatory activity as well as one β_2_AR agonist, ephedrine, were identified [23]. Although the bronchodilation effect of QFXY could be partially blocked by a classicalβ receptor antagonist, propranolol (Pro), the smooth muscle relaxant effect of QFXY exceeds that of an identical dose of ephedrine, possibly because the biological activities of QFXY result from a mixture of active compounds rather than from ephedrine alone. A mutually enhanced bioactivity-guided UPLC/Q-TOF-MS characterization system for β_2_AR-agonist cofactors was proposed in this paper to determine the rationale for the formula of QFXY. Several ingredients that have synergistic effects with ephedrine on the β_2_AR/cAMP signal pathway were suggested, and the efficacies and mechanisms were tested.

## Materials and Methods

### 1. Reagents and Materials

HPLC-grade solvents were purchased from Tedia (Fairfield, CA, USA). Deionized water was purified using a Milli-Q system (Millipore Laboratory, Bedford, MA, USA). Commercial QFXY (lot no. 5230139) was purchased from Darentang Pharmaceutical Company (Tianjin, China). Ephedra herbal samples (lot no. 1105139131) were purchased from Anguo Changan Limited Company (Anguo, Hebei, China). A standard arctigenin (Atg) sample was obtained from Tianjin Institute of Pharmaceutical Research (Tianjin, China). Ephedrine hydrochloride was purchased from Yifang S&T (Tianjin, China). Salbutamol was purchased from Sigma Chemical Co. (St. Louis, MO, USA). Fluo-4, in the form of acetoxymethyl ester (Fluo-4/AM), and Lipofectamine 2000, a transfection reagent, were purchased from Invitrogen (Carlsbad, CA, USA). Two Luc2p reporter plasmids, pGL4.29 and pGL4.30, and a Renilla luciferase reporter vector, pRL-TK plasmid, were obtained from Promega (Madison, WI, USA). The β_2_AR-transfected human embryonic kidney 293 (β_2_AR-HEK 293) cells were grown in our laboratory [24]. Hanks' balanced salt solution (HBSS) (Ca^2+^ and Mg^2+^ free), Dulbecco's modified Eagle medium (DMEM), and fetal bovine serum (FBS) reagents for the cell culture were purchased from HyClone (UT, USA). The remaining reagents were of analytical grade.

### 2. Sample Preparation and UPLC Separation

The ephedra herbal samples (100 g) were powdered and soaked in 1 L of 0.1 mol/L HCl overnight; after a 30-min ultrasonic treatment, the supernatant was filtered and adjusted to a pH of 11. Subsequently, the samples were passed through a column (2×20 cm) containing D151 macroporous resin and eluted with 0.1 mol/L HCl. The eluate was freeze-dried to provide 0.388 g of ephedra extract (EE). QFXY (1 g) were crushed and dissolved in 10 ml of methanol under ultrasonic conditions. After passage through a 0.22-µm filter, the filtrate was frozen at −20°C for 12 hours and centrifuged at 13,000 rpm for 10 min at 4°C to remove the excipient polyethylene glycol precipitation; the supernatant was used for the analysis.

### 3. Ethics Statements

The animal experiments were strictly performed under the guide lines for the treatment of laboratory animals of Nankai University, and the animal protocols were approved by the Institute Research Ethics Committee at Nankai University. To minimize animal suffering, the test animals were sacrificed by cervical dislocation after the experiments.

### 4. Histamine-Induced Asthma Model in Guinea Pigs

A histamine (His)-induced guinea pig asthma model was developed using standard methods [25]. Thirty male Hartley strain guinea pigs (approximately 300 g) with identical latent periods of His-stimulated asthma were divided into 6 groups during the preliminary experimental period. During the experiment, the animals were kept in an inhalation cage consisting of 3 boxes. An animal was placed into box A for drug administration using an ultrasonic nebulizer to atomize the test drug solutions. The drug was atomized at 0.5 ml/min and disseminated into box A. The animal was kept in box A for 1 min under spontaneous breathing during the administration of the atomized test drugs or normal saline. Box B served as a sluice through which the animal was passed into box C. In box C, the animal was exposed to an atomized 0.1% His solution for 20 s. His was atomized at 0.5 ml/min and disseminated into box C. The animal was subsequently withdrawn from the inhalation cage. The time that elapsed until the appearance of an asphyxial convulsion was considered the latent asthma period. The increasing rate of the latent period was calculated using Vogel's method, and the response of the pre-experimental group was recorded as 100%. The rate of increase in the latent period (%) for the test group was used to evaluate the activity.

### 5. The Relaxant Test on the Guinea Pig Tracheal Muscle

In vitro spasmolytic activity tests on isolated tracheas were conducted, as previously described, with a slight modification [26]. The tracheal strips were mounted vertically in a 20-ml water-jacketed organ bath filled with Krebs-bicarbonate buffer under 95% O_2_ and 5% CO_2_ at 37°C. Before each experiment, each strip was subjected to a 1 g load for at least 1 h with frequent changes of the Krebs-bicarbonate buffer until a stable baseline tension was obtained. The strips were washed thoroughly with Krebs-bicarbonate buffer immediately after the peak tension developed and remained unstimulated until a stable baseline tension was obtained. In every experiment, 200 µL of each drug was added to the organ bath, and all of the drug concentrations were expressed as the final concentration; then the test samples were added at 10 min per series of acetylcholine (Ach) concentrations. The peak contractile response was recorded as 100%. The half-maximal effective concentration values of Ach for the tensions were expressed as 50% of the peak contractile response (EC50) and were used to evaluate the activity.

### 6. UPLC/Q-TOF Analysis

A Waters Acquity UPLC instrument system (Waters Co., USA) equipped with a photo diode array detector (DAD) (190–400 nm) was used for the analysis, and the system was controlled by MassLynx V4.1 software (Waters Co., USA). The separations were performed using a Waters Acquity BEH C_18_ column (2.1 mm×100 mm, 1.7 µm). A gradient elution of 1% formic acid solution (A) and CH_3_CN (B) was performed as follows: 2% B maintained from 0–10 min, 10–40% B from 10–15 min, 40–95% B from 15–26 min, and 95–100% from 26–35 min. The flow rate was 0.40 ml/min, and the column temperature was 30°C. The injection volume was 1.0 µL. The UPLC effluent was split 1∶9 after the substance separation. The 10% fraction was directed toward the Q-TOF-MS for the structural analysis. The 90% fraction was directed toward the diode array detector and was collected into a 96-deep-well plate (2.2 ml) every 0.5 min. After being evaporated to dryness in a 40°C vacuum drying oven, the residues were dissolved in cell culture medium (100 µL) for the luciferase reporter activity assay.

Accurate mass measurements and MS/MS were performed using a Waters Q/TOF Premier with an ESI system (Waters, Manchester, UK). The ESI-MS spectra were acquired in the negative and positive ionization modes. The capillary voltage was 3.0 kV for the negative mode and 2.5 kV for the positive mode. The sample cone voltage during the positive ion mode was set to 30 V, whereas that during the negative ion mode was set to 45 V; high-purity nitrogen was used as the nebulization and auxiliary gas. The nebulization gas was set to 600 L/h at 350°C, the cone gas was set to 50 L/h, and the source temperature was 100°C. The Q/TOF Premier acquisition rate was 0.1 s with a 0.02-s inter-scan delay. Argon was utilized as the collision gas at 5.3×10^−5^Torr. The instrument was operated with the first resolving quadrupole in the wide-pass mode (50–1200 Da), whereas the collision cell operated at two alternative energies (i.e., 5 and 30 eV). Leucine encephalin amide acetate was used as the lock mass ([M−H]^−^ = 553.2775, [M+H]^+^ = 555.2931) at a concentration of 200 pg/µL and was added at 20 µL/min.

### 7. Luciferase Reporter Assay

The β_2_AR-HEK 293 cells were grown in Dulbecco's modified Eagle's medium (DMEM) (Gibco BRL) containing 10% fetal bovine serum (FBS) (Gibco BRL), 100 U/ml penicillin and 0.1 mg/ml streptomycin at 37°C and 5% CO_2_. For the β_2_AR/cAMP activity analysis, the culture medium was replaced after 24 h, and the cells were co-transfected with 100 ng/well pCRE-Luc reporter plasmid pGL4.29 and 16 ng/well Renilla luciferase reporter vector pRL-TK plasmid, as previously described [27]. For the intracellular calcium concentration detection, the β_2_AR-HEK 293 cells were co-transfected with 100-ng/well pGL4.30 Luc plasmid and 10 ng/well Renilla luciferase reporter vector pRL-TK plasmid, according to the manufacturer's protocol. After 24 h of transfection, the cells were then pretreated with different drugs and stimulated by ionomycin (1 mmol/L) combined with 12-myristate 13-acetate (PMA, 1 mg/ml) for 9 h for the cell bioactivity assay. The cells were washed, lysed, and assayed for luciferase activity using a dual-luciferase reporter assay system (Promega) according to the manufacturer's instructions. The relative luciferase activity was obtained by normalizing the firefly luciferase activity against the activity of the internal Renilla luciferase control (Modulus, Turner BioSystems, USA). The ratio of the firefly luciferase activity to the renilla luciferase activity was used to normalize the differences in the transfection efficiency.

### 8. Apparent Embodiment of Intracellular Ca^2+^

Human bronchial smooth muscle cells (HBSMC) were obtained from Sciencell (cat. #3400) and cultured in DMEM supplemented with 10% FBS. The HBSMCs were loaded with Ca^2+^ indicator dye Fluo-4/AM according to the manufacturer's instructions. The cells were incubated in 1 ml of HBSS (Ca^2+^ and Mg^2+^ free) containing 5 µM Fluo-4/AM for 30 min at 37°C. After loading, to de-esterify the Fluo-4/AM, the cells were washed with HBSS to remove excess dyes and then equilibrated for 30 min. The changes in the fluorescence intensity of the Fluo-4-Ca^2+^ complex were monitored by confocal microscopy (TCS SP5, Leica, Germany). The stained cells were treated with samples immediately before the confocal microscopy, and the intracellular [Ca^2+^]i changes at 494/516 nm (Ex/Em) were recorded. The [Ca^2+^]i curve was analyzed in the single-cell mode.

### 9. Statistical Analysis

The results are expressed as the standard error of the mean (SEM). Multiple comparisons were performed using ANOVA, followed by Bonferroni's post hoc test. For single comparisons, the significant differences between the means were determined using Student's *t*-test. *P*<0.05 was considered statistically significant.

## Results and Discussion

### 1. The Bronchodilator Effect of QFXY

**Figure 1:**
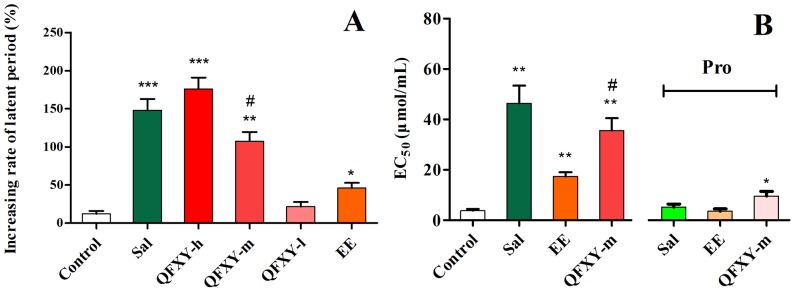
The bronchodilator effect of QFXY on an atomized His-induced guinea pig asthma model (A) and the guinea pig tracheal muscle relaxant test (B). The values are presented as the mean ± SEM (*n* = 5). *P<0.05, **P<0.01, ***P<0.001, compared to the control group; ^#^ *P*<0.05, middle dose QFXY compared to the EE group.

The guinea pig asthma model was established to validate the bronchodilation effect of high, middle and low doses of QFXY (397.5, 132.5, and 44.1 mg/100 g, respectively) and EE (0.146 mg/100 g)which containing the same dose of ephedrine (about 21.9 µg) with the middle dose QFXY. As shown in Figure 1A, 10^−6^ mol/L salbutamol (Sal) used as a positive control prolonged the latent period by approximately 1.48-fold. QFXY inhibited asphyxial convulsions in a concentration- dependent manner. The increasing rates of the high, middle and low doses were approximately 1.76-, 1.07-, and 0.22-fold, respectively. The EE contained the identical dose of ephedrine as the middle dose of QFXY and prolonged the latent period only 0.46-fold. Additionally, the identical effect was observed on the guinea pig tracheal muscle relaxant test, and the EC_50_ of the middle QFXY dose was more effective than the EE extract (Figure 1B). Differing from Sal and the EE extract, the relaxant effect of QFXY was not completely inhibited by 10^−7^ mol/L Pro. As a result, QFXY was more effective for reducing the severity of bronchoconstriction after exposing His or Ach stimulation than when the ephedrine extract was used alone. In addition to the monarch drug (Herba Ephedrae), other ingredients played a role in the anti-asthmatic action.

### 2. Screening Synergistic Bronchodilators from QFXY

**Figure 2:**
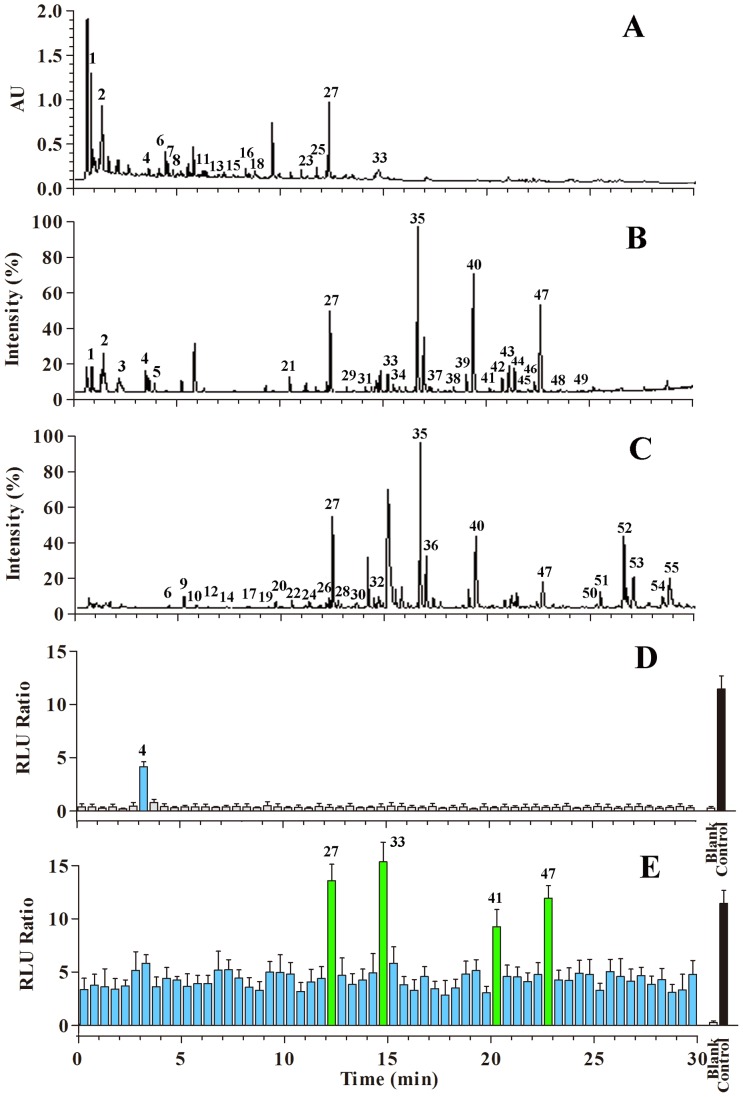
UPLC/Q-TOF-MS and synergic β_2_AR activation-bioactivity analysis of QFXY. (A) The UPLC/UV chromatograms of QFXY observed at 254 nm; (B, C) The TIC chromatograms in (B) the positive and (C) negative ESI modes; (D) The bioactivity chromatograms obtained using the dual-luciferase reporter assay system for β_2_AR activation; (E) The mutually enhanced bioactivity chromatograms obtained using the dual-luciferase reporter assay system for β_2_AR activation.

The optimal UPLC conditions were applied for purifying the components of QFXY (Figure 2A). The total ion current chromatograms collected in the positive and negative ESI modes are shown in Figure 2B and 2C, respectively. The [M+H]^+^ and [M−H] ^−^ ions were obtained with as much relevant information as possible to confirm the molecular weight and structure of the constituents. Finally, 55 compounds were identified in QFXY (the data are not shown). As shown in Figure 2D, only peak no. 4 (ephedrine, Eph) has been identified as a β_2_AR agonist; the synergistic ingredient for bronchodilation remains unknown.

To reveal the cofactors, a modified system incorporating a dual-luciferase reporter validation system was established. After incubation with every UPLC effluent sample for 3 hours, the identical dose of Eph (10^−5^ mol/L) was added to each well; the samples were incubated 3 hours and subjected to the luciferase reporter activity assay. Consequently, four fractions (corresponding peak no. 27, 33, 41 and 47) with significant synergistic effects for the β_2_AR agonist were identified (Figure 2E). Peak 47 (22.425 min) was selected to illustrate the identification approach. The base peak in the positive ESI mode was *m/z* 403.1401 and was confirmed to be [M+H]^+^. The elemental and possible molecular compositions were deduced using the exact molecular weight. These molecular compositions were assessed using Ref. [28], and only descurainolide B (C_21_H_22_O_8_) was revealed as the most probable compound from Lepidii Semen. The other constituents were identified as arctiin and Atg (from Arctii Fructus) as well as descurainoside (from Lepidii Semen) using the identical approach; the detailed fragment information is listed in Table 1.

**Table 1:** The MS/MS data in both ESI modes and the identification of the compounds in QFXY possessing synergic effects on the β_2_AR-signaling pathway.

Peak No.	*t*_R_(min)	Identification	Mode	*m*/*z*	MS^2^	Composition	Herb
4	3.985	Ephedrine	Pos	166.1235	331[2M+H]^+^,166[M+H] ^+^,148[M+H-H_2_O]+,117[M+H-H_2_O-NHCH_3_]^+^	C_10_H_15_NO	EH
27	12.444	Arctiin	Pos	535.2139	535[M+H]^+^,491[M+H-CO_2_]^+^,373[M+H-Glu]^+^,339[M+H-Glu-2OH]^+^	C_27_H_34_O_11_	AF
33	15.256	Arctigenin	Pos	373.1644	373[M+H]^+^,355[M+H-H_2_O]^+^,337[M+H-2H_2_O]^+^,324[M+H-H_2_O-OCH_3_]+	C_21_H_24_O_6_	AF
41	20.412	Descurainoside	Pos	401.0911	401[M+H]^+^,383[M+H-H_2_O]^+^,365[M+H-2H_2_O]^+^,333[M+H-2H_2_O-CH_3_OH]^+^	C_17_H_20_O_9_S	LS
47	22.425	Descurainolide B	Pos	403.1401	403[M+H]^+^,371[M+H-CH_3_OH]^+^,353[M+H-CH_3_OH-H_2_O]^+^,321[M+H-2CH_3_OH-H_2_O]^+^	C_21_H_22_O_8_	LS

**Figure 3:**
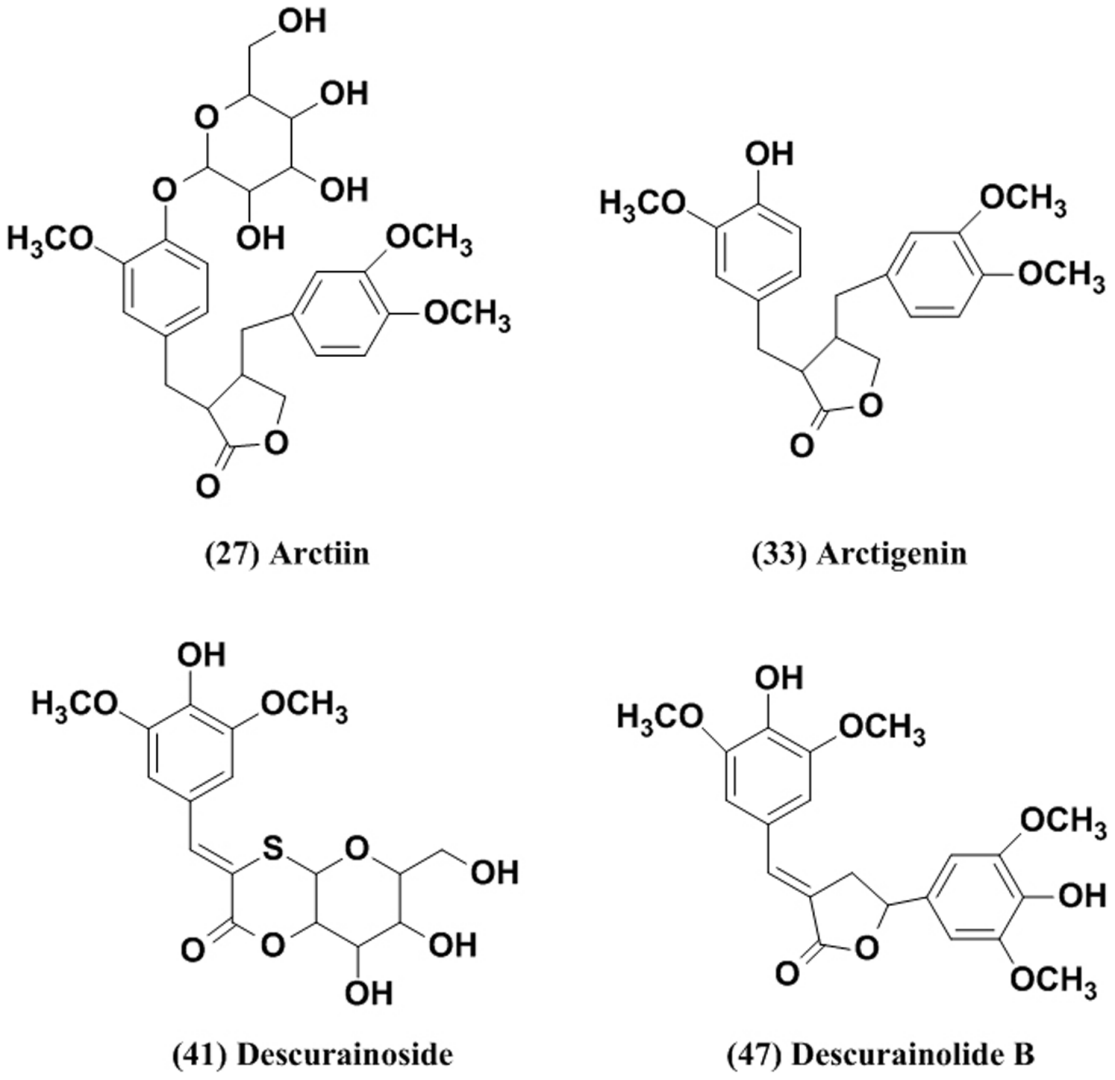
Structures of the QFXY constituents exhibiting a synergic effect on the β_2_AR-signalling pathway.

*Descurainia Sophia* L. is widely distributed in northeastern China, and its seeds (Lepidii Semen) are used to relieve coughing, prevent asthma, reduce edema, promote urination and induce a cardiotonic effect. Biological screening of the alcoholic extract revealed that the plant is highly safe and has analgesic, antipyretic and anti-inflammatory effects [29]. Arctiin and its aglucone, Atg, are found in the fruits of *Arctiumlappa* L (ArctiiFructus). Arctiin has anti-inflammatory, anti-oxidant, antibacterial, and antiviral effects in vitro [30], [31]. The plasma pharmacokinetics and tissue distribution of arctiin and its major metabolite Atg in rats have been validated [32]. Recently, Atg was used as an antitumor agent that killed tumor cells via glucose deprivation by inhibiting cellular energy metabolism [33], [34]. The four ingredients were lignin compounds identified from two herbs (Figure 3). There has been a lack of studies regarding the effects of these compounds on β_2_AR agonist synergistic activity.

### 3. The Synergistic Effect of Eph and Atg on the cAMP Response and Tracheal Muscle

**Figure 4:**
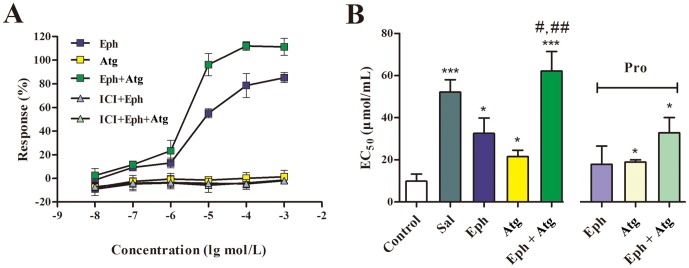
The synergistic effects of Eph and Atg on β_2_AR activation evaluated by the dual luciferase reporter assay system (A) and the guinea pig tracheal muscle relaxant test (B). Each bar represents the mean ± SEM, *n* = 5 per group, and the EC_50_ values are expressed as the mean. *P<0.05, **P<0.01, ***P<0.001, compared to the control group; ^#^ *P*<0.05, compared to the Eph group; ^##^ *P*<0.01, compared to the Atg group.

In this study, Atg was selected to validate its synergistic effect with the β_2_AR agonist using a dual-luciferase reporter assay for the β_2_AR/cAMP signal pathway. As shown in Figure 4A, Atg alone did not exhibit activity. However, compared to Eph alone, Atg could significantly enhance the cAMP response of ephedrine by shifting the dose-response curve with the EC_50_ value from 1.463 to 9.331 mol/L. ICI 118551 (10 µmol/L), a selective β_2_AR inhibitor, could completely block Eph plus Atg and Eph alone. The cAMP synergistic effect of Atg on Eph was β_2_AR dependent.

The *in vitro* tracheal muscle relaxant test, treatment with 10^−5^ mol/L Eph or Atg, significantly inhibited the tracheal contractions induced by a series of ACh concentrations in a dose-dependent manner, and the EC_50_ values were 32.6 and 21.5 µmol/L, respectively (Figure 4B). When combined with the above-mentioned dose of Eph and Atg, the EC_50_ value increased to 62.1 µmol/L and exceeded that of the 10^−8^ mol/L Sal positive control group (52.1 µmol/L). Blocking the β_2_ARs with 10^−7^ mol/L Pro significantly affected the inhibitory effect exerted by an identical dose of Eph; however, there was little effect on the Atg or Atg plus Eph (32.8 µmol/L). Atg could inhibit tracheal contractions independently; however, the synergistic relaxant effect was based on the β_2_AR/cAMP pathway.

### 4. The Synergistic Mechanism of Atg in Reducing Intracellular Calcium Concentration

**Figure 5:**
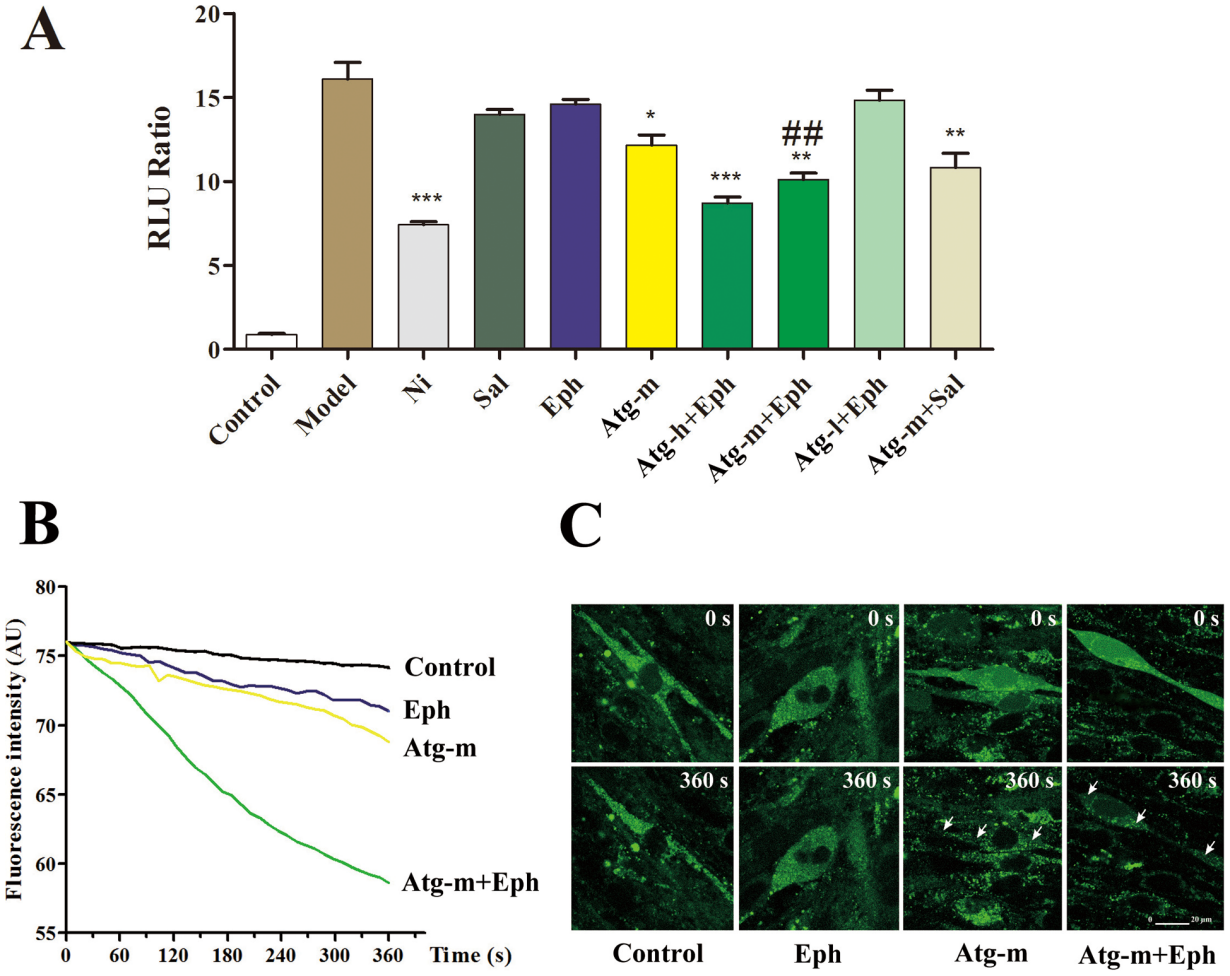
The synergistic mechanism of Atg and Eph in reducing the intracellular calcium concentration, asevaluated by the dual luciferase reporter assay system (A), confocal microscopy fluorescence intensity analysis (B) and image observations (C). The white arrowindicates a significant [Ca^2+^]_i_ intensity decrease in a singleHBSMCcell. The values are presented as the mean ± SEM (*n* = 5). *P<0.05, **P<0.01, ***P<0.001, compared to the model group (M); ^##^ *P*<0.01, compared to the Eph group.

The major pathway that mediates airway smooth muscle constriction is the activation of phospholipase C, with the release of inositol 1,4,5-triphosphate and the elevation of intracellular calcium levels [35]. Ach and His could increase the intracellular free Ca^2+^ by the activation of the muscarinic M3 receptor and the histamine H1 receptor and a phospholipase C-dependent mechanism [36]. Complex cAMP and calcium crosstalk occurs between these pathways and leads to the careful regulation of airway smooth muscle tone [37]; however, cytoplasmic Ca^2+^ concentrations [Ca^2+^]_i_ and myosin light chain phosphorylation are considered key elements [38]. It was reported recently that Atg could regulate human bronchial smooth muscles by affecting transmembrane Ca^2+^ flow [39]. In this paper, the crosstalk between a β_2_AR agonist with intracellular free Ca^2+^ was investigated. Compared with treatment with10^−8^ mol/L Sal or 10^−5^ mol/L Eph, 10^−5^ mol/L Atg-m reduced the intracellular free calcium concentration; however, the reduction was less than that by the calcium channel blocker nimodipine (10^−5^ mol/L) (Figure 5A). When Eph (10^−5^ mol/L) combined with high, middle and low doses of Atg (10^−4^, 10^−5^ and 10^−6^ mol/L, respectively), the intracellular calcium levels decreased in a dose-dependent manner. The identical effect was observed in the Sal combination group. The cytosolic [Ca^2+^]_i_ components of the HBSMCs were detected with confocal microscopy. As shown in Figure 5B, compared with the control or Eph (10^−5^ mol/L) group, Atg-m (10^−5^ mol/L) clearly promoted Ca^2+^ efflux during a time interval of 360 s. In the Atg-m plus Eph group, the [Ca^2+^]_i_ changed more obviously; [Ca^2+^]_i_ intensity images in the single-cell mode treated with Atg-m and Eph at 0 s and 360 s are shown in Figure 5C and present a significant decrease. The mechanism of Atg as a synergistic bronchodilator that exhibited a relaxation effect in the airway smooth muscle by reducing the intracellular free calcium was clarified.

## Conclusions

The contractile and relaxant responses of airway smooth muscle are regulated by crosstalk between the important intracellular signaling pathways controlling [Ca^2+^]_i_ and cAMP. For over ten years, evidence-based guidelines for COPD or asthma have recommended β_2_AR agonists as the principal agents for maintenance pharmacotherapy. In this paper, four lignin ingredients, arctiin, Atg, descurainoside and descurainolide B, which demonstrated synergistic smooth muscle relaxant effects with ephedrine dependent on the β_2_AR/cAMP signal pathway, were identified from the QFXY prescription. The mechanism of Atg as a β_2_AR agonist cofactor that could reduce intracellular free calcium was proposed. Additionally, our data indicated the β_2_AR agonist, ephedrine, as the principal bronchodilator of the QFXY formula, whereas the lignin ingredients that regulated [Ca^2+^]_i_ served as adjuvant components. A greater understanding of the mechanisms governing the control of these pathways based on conventional wisdom could lead to the discovery of novel therapeutic regimens, which could yield novel agents for the treatment of COPD or asthma.
